# Predictive Value of Epicardial Adipose Tissue Thickness for Plaque Vulnerability in Left Coronary Arteries: Histological Evidence from 245 Sudden Cardiac Death Cases

**DOI:** 10.3390/diagnostics15121491

**Published:** 2025-06-11

**Authors:** Raluca Niculescu, Alexandru Mureșan, Carmen Corina Radu, Timur Robert Hogea, Iuliu Gabriel Cocuz, Adrian Horațiu Sabău, Eliza Russu, Emil Marian Arbănași, Eliza Mihaela Arbănași, Adrian Vasile Mureșan, Adina Stoian, Daniela Edith Ceană, Corneliu Florin Buicu, Ovidiu Simion Cotoi

**Affiliations:** 1 Department of Pathophysiology, George Emil Palade University of Medicine, Pharmacy, Science and Technology of Targu Mures, 540139 Targu Mures, Romania; raluca.niculescu@umfts.ro (R.N.); iuliu.cocuz@umfst.ro (I.G.C.); adrian-horatiu.sabau@umfst.ro (A.H.S.); adina.stoian@umfst.ro (A.S.); ovidiu.cotoi@umfst.ro (O.S.C.); 2 Department of Pathology, Mures Clinical County Hospital, 540011 Targu Mures, Romania; 3 Doctoral School of Medicine and Pharmacy, George Emil Palade University of Medicine, Pharmacy, Science and Technology of Targu Mures, 540139 Targu Mures, Romania; emil.arbanasi@umfst.ro (E.M.A.); arbanasi.eliza@gmail.com (E.M.A.); 4 Clinic of Vascular Surgery, Mures County Emergency Hospital, 540136 Targu Mures, Romania; alex.muresan99@yahoo.ro; 5 Department of Forensic Medicine, George Emil Palade University of Medicine, Pharmacy, Science and Technology of Targu Mures, 540139 Targu Mures, Romania; hogea.timur@umfst.ro; 6 Institute of Forensic Medicine, 540141 Targu Mures, Romania; 7 Department of Vascular Surgery, George Emil Palade University of Medicine, Pharmacy, Science and Technology of Targu Mures, 540139 Targu Mures, Romania; eliza.russu@umfst.ro (E.R.); adrian.muresan@umfst.ro (A.V.M.); 8 Regenerative Medicine Laboratory, Centre for Advanced Medical and Pharmaceutical Research (CCAMF), George Emil Palade University of Medicine, Pharmacy, Science and Technology of Targu Mures, 540139 Targu Mures, Romania; 9 Department of Public Health, George Emil Palade University of Medicine, Pharmacy, Science and Technology of Targu Mures, 540139 Targu Mures, Romania; daniela.ceana@umfst.ro (D.E.C.); florin.buicu@umfst.ro (C.F.B.)

**Keywords:** epicardial adipose tissue (EAT), sudden cardiac death (SCD), unstable plaque, biomarkers, autopsy

## Abstract

**Background/Objectives:** Cardiovascular disease remains the leading global cause of death, with atherosclerotic plaque vulnerability, rather than stenosis severity, playing a central role in acute coronary events. Epicardial adipose tissue (EAT) has emerged as a key contributor to coronary atherosclerosis and myocardial ischemia. This study aimed to investigate the relationship between EAT thickness and the development and severity of atherosclerotic plaques in these coronary arteries, and to evaluate the influence of demographic factors on EAT thickness and plaque vulnerability. **Methods**: A retrospective analysis was conducted on autopsy data from 245 sudden cardiac death (SCD) cases (2021–2023). EAT thickness was measured at the left anterior descending artery (LAD) and left circumflex coronary artery (LCx) levels. From each artery, one segment that showed evidence of an atherosclerotic plaque was collected and sent for histological examination. Additionally, we documented demographic data, including age, sex, and body mass index (BMI) for each case. **Results**: In the present study, we enrolled 245 subjects with SCD, among whom 175 (71.42%) were male, and 70 (28.58%) were female. The mean age was 62.31 ± 12.69 years, and the mean BMI was 26.12 ± 4.16. We observed a mean EAT thickness value of 0.74 ± 0.26 cm at the LAD artery level and 0.71 ± 0.27 cm at the LCx artery level. We observed a positive correlation between BMI and EAT thickness at the LAD level (r = 0.260, *p* < 0.001) and similarly at the LCx level (r = 0.260, *p* < 0.001). Additionally, advancing age is associated with an increase in EAT thickness at both the LAD level (r = 0.188, *p* = 0.003) and the LCx level (r = 0.242, *p* < 0.001). Furthermore, we observed a higher EAT thickness at the LAD level (*p* = 0.0019) and the LCx level (*p* = 0.0225) among subjects with unstable atherosclerotic plaques. In the logistic regression analysis, the elevated value of EAT thickness was associated with unstable atherosclerotic plaque at LAD (OR: 1.88, *p* = 0.002) and LCx (OR: 1.51, *p* = 0.010) for the entire study cohort. **Conclusions**: Our data revealed that higher baseline values of EAT LCx and EAT LAD are associated with unstable plaque at the level of the left coronary arteries. Furthermore, our findings indicate that male individuals are more susceptible to developing unstable plaques in the coronary arteries.

## 1. Introduction

Cardiovascular disease (CVD) remains the leading cause of mortality worldwide, accounting for over 17 million deaths annually [1,2]. A significant portion of these deaths is attributed to myocardial infarction (MI), most caused by atherosclerosis [3]. The atheromatous plaque formation begins early in life and progresses as lipid accumulation and inflammation persist within the vessel walls, ultimately leading to more unstable plaque forms [4,5]. However, the vulnerability of atherosclerotic plaque significantly influences the occurrence of acute coronary events more than the extent of stenosis [4,6]. Consequently, considerable attention has been devoted in recent years to characterizing plaque morphology, the inflammatory activity present within the epicardial adipose tissue (EAT), and the implications of these factors on the progression of atherosclerotic plaque [4,6,7,8,9].

Recent studies have highlighted the critical role of EAT in the initiation and progression of coronary atherosclerosis, obstructive coronary artery disease (CAD), myocardial ischemia, and major adverse cardiac events (MACE) [7,8,9]. Nonetheless, EAT protects against hypothermia and mechanical stress under optimal conditions, thereby supplying myocardial energy [9]. It affects coronary artery health through mechanisms such as inflammation, oxidative stress, immune responses, and lipid accumulation [9,10,11]. This occurs via the secretion of both anti-inflammatory and pro-inflammatory adipokines, with the pro-inflammatory adipokines being more predominant in conditions characterized by excessive EAT. Such dynamics contribute to the dysfunction of coronary endothelium and the progression of the disease [9,10,11].

The distribution of EAT is not uniform throughout the heart, leading to regional effects, particularly around the proximal coronary arteries due to their anatomical proximity to pericoronary EAT [10,12,13]. Clinically, increased EAT volume and thickness have been linked to a higher burden of atherosclerotic plaques, significant coronary stenosis, and an elevated risk of myocardial ischemia and MACE [7,8,9,12,13]. These associations are well documented in middle-aged symptomatic individuals, where EAT volume correlates with high-risk plaque features such as low-attenuation non-calcified plaque [14]. Recently, Timur et al. [15,16] have demonstrated that elevated values of EAT are associated with sudden cardiac death (SCD) and are predictive of silent myocardial infarction, as evidenced by two autopsy studies. However, the role of EAT in the different stages of atherosclerosis, particularly in young adults and asymptomatic individuals, remains largely unexplored.

This study aims to examine the impact of coronary epicardial adipose tissue thickness on the development and severity of atheromatous plaques at the level of the left anterior descending artery and the left circumflex artery. Secondly, we will explore how demographic factors influence EAT thickness and the related risk of unstable atherosclerotic plaques in the left coronary arteries. The results could offer important insights into its role as an early diagnostic marker and risk stratification tool.

## 2. Materials and Methods

### 2.1. Study Design

In the present study, information obtained from autopsies of subjects with SCD performed at the Department of Forensic Medicine in Târgu Mureș between January 2021 and December 2023 was analyzed. Patients under the age of 18 were excluded from the analysis due to the lack of atherosclerotic deposits and the high variability of anthropometric characteristics. Furthermore, cases characterized by advanced decomposition, pericardial tamponade, perforated pericardium, and fatalities in which toxicology reports revealed the presence of alcohol, drugs, illicit substances, poisons, or other chemical compounds in lethal concentrations were excluded owing to the inadequacy of the determinants of EAT thickness, as well as the morphology of coronary atherosclerotic plaque. Additionally, the analysis excluded cases involving coronary stents or bypass procedures due to their significant alteration of the vascular architecture. Subjects whose deaths were classified as violent, specifically suicide, road accidents, electrocution, or incineration, were excluded from the analysis. Finally, 245 individuals were enrolled, from whom all characteristics were easily analyzed, with 175 (71.42%) being men and 70 (28.58%) being women.

### 2.2. Autopsy Characteristics

The medico-legal autopsy was conducted in accordance with established protocols [15,16] and guidelines [17,18,19], and began with a comprehensive external examination of the body, including the recording of height, weight, and calculation of the body mass index (BMI). A systematic dissection of all anatomical cavities is performed in a cranio-caudal direction. In all cases of SCD, a cardiac cause is considered only after the exclusion of non-cardiac etiologies, including cerebral, respiratory, gastrointestinal, or hemorrhagic/septic shock origins. Following thoracic cavity opening and documentation of pleural contents and findings, the thoraco-cervico-buccal block (including the tongue, esophagus, trachea, heart, and lungs) is excised en bloc and examined in anatomical orientation [15,16,17,18,19]. The pericardial sac is assessed for integrity and opened using an inverted Y incision. The pericardial cavity is inspected for effusion, hemorrhagic collections, adhesions, or neoplastic infiltrates.

The major blood vessels undergo anatomical inspection, and the heart is excised through a series of transverse cuts. The arterial cut occurs at least 3 cm beyond the aortic and pulmonary valves, followed by a venous cut at least 2 cm from where the superior vena cava joins the right atrial appendage, and another venous cut 2 cm from where the pulmonary veins enter the left atrium. The outflow tracts of the aorta and pulmonary arteries, along with the respective valves, are opened and examined down to their insertions. The coronary arteries are carefully dissected from their origin all along their path using successive transverse incisions. The thickness of EAT is measured 1 cm from the coronary ostia, and specimens of the coronary arteries are collected for histological study. The arterial specimen of the left anterior descending artery (LAD) and left circumflex coronary artery (LCx), along with the surrounding EAT and at least 0.5 cm of the adjacent myocardial tissue, is fixed en bloc [15,16].

### 2.3. Histopathological Characterization of Coronary Atherosclerotic Plaque

Coronary artery samples collected during autopsy underwent a series of histopathological preparation steps. Initially, the tissue fragments were fixed in 4% formaldehyde for a minimum of 24 h. Following fixation, an automated tissue processor was used to prepare the samples into paraffin blocks. Thin sections were then cut from these paraffin-embedded samples and stained using the standard hematoxylin and eosin method. Two medical examiners independently assessed and scored gross pathological findings observed during autopsies. The extent of coronary atherosclerosis was determined through histopathological examination, in accordance with the histological classification system established by the American Heart Association’s Committee on Vascular Lesions of the Council on Arteriosclerosis [20]. This grading system includes the following lesion types: initial lesion (type I), progression-prone lesion (type II), pre-atheroma or intermediate lesion (type III), atheroma (type IV), fibroatheroma (type Va), calcified lesion (type Vb), fibrotic lesion (type Vc), and advanced lesion with surface defect, hemorrhage, hematoma, or thrombotic deposit (type VI).

### 2.4. Statistical Analyses

The statistical analysis was conducted using SPSS for Mac OS version 29.0.2.0 (SPSS, Inc., Chicago, IL, USA). Chi-square tests were employed to evaluate the differences between male and female subjects concerning the type of atherosclerotic plaque present in the left coronary arteries. In addition, Student’s *t*-test or Mann–Whitney tests were utilized to assess the differences in continuous variables. To analyze the predictive power and establish the cut-off values of BMI, EAT LCx, and EAT LAD, a receiver operating characteristic (ROC) curve analysis was performed. This ROC curve analysis facilitated the determination of appropriate cut-off values for the previously mentioned variables based on Youden’s index. To ascertain the predictive role of EAT left circumflex coronary artery (LCx) and EAT left anterior descending artery (LAD), three adjustment models were proposed in the multivariate analysis: Model 1 (unadjusted), Model 2 (adjusted for age and sex), and Model 3 (adjusted for age, sex, and BMI). Furthermore, multivariate analysis was conducted separately for the subgroup of female subjects and the subgroup of male subjects.

## 3. Results

In the present study, we enrolled 245 subjects with SCD, among whom 175 (71.42%) were male, and 70 (28.58%) were female. The mean age was 62.31 ± 12.69 years, and the mean BMI was 26.12 ± 4.16 (Table 1). We observed a mean EAT thickness value of 0.74 ± 0.26 cm at the LAD artery level and 0.71 ± 0.27 cm at the LCx artery level. Additionally, we noted that 205 subjects (83.57%) exhibited unstable atherosclerotic plaques at the LAD level and 181 subjects (73.88%) at the LCx level (Table 1). Regarding the difference between sexes, we observed a higher age (*p* < 0.001) and an increased BMI (*p* = 0.044) in female subjects, without differences in EAT thickness in the LAD (*p* = 0.604) or LCx (*p* = 0.377) (Table 1). Additionally, female subjects exhibited a higher prevalence of type IV atherosclerotic plaques (28.57% vs. 15.42%, *p* = 0.018) and a lower incidence of type VI plaques (4.29% vs. 14.29%, *p* = 0.026) in the LAD compared to their male counterparts. Conversely, male subjects demonstrated a greater incidence of unstable atherosclerotic plaques in both the LAD (*p* = 0.001) and LCx (*p* = 0.03) (Table 1).

**Figure 1:**
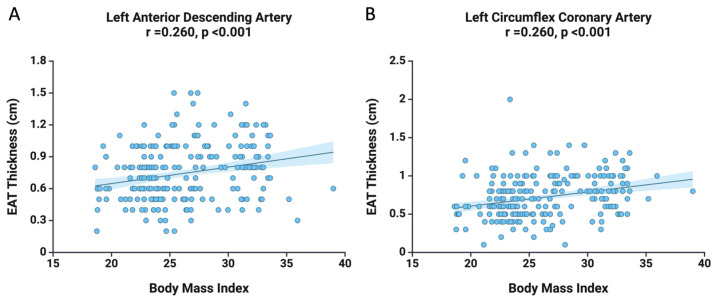

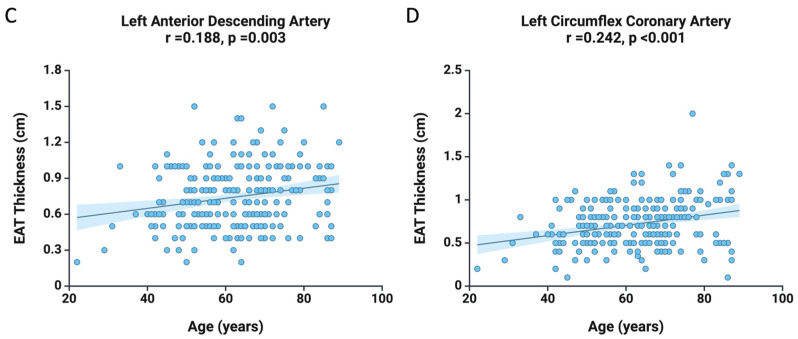
The correlation between BMI and EAT thickness at the level of LAD (**A**) and LCx (**B**), as well as the correlation between age and EAT thickness at the level of LAD (**C**) and LCx (**D**).

Moreover, we aimed to examine the variables correlated with EAT thickness at the LAD and LCx levels. As illustrated in Figure 1, we observed a positive correlation between BMI and EAT thickness at the LAD level (r = 0.260, *p* < 0.001) and similarly at the LCx level (r = 0.260, *p* < 0.001). Additionally, advancing age is associated with an increase in EAT thickness at both the LAD level (r = 0.188, *p* = 0.003) and the LCx level (r = 0.242, *p* < 0.001).

**Figure 2:**
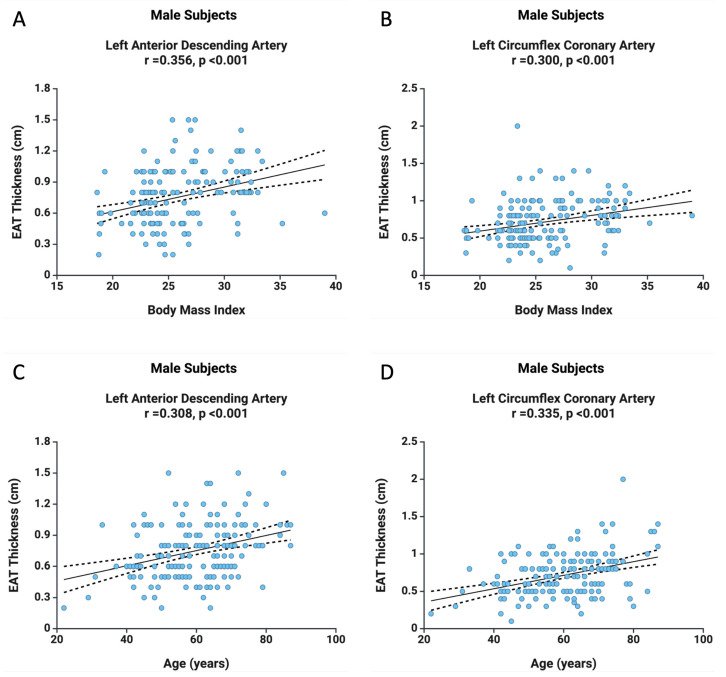
The correlation between BMI and EAT thickness at the level of LAD (**A**) and LCx (**B**), as well as the correlation between age and EAT thickness at the level of LAD (**C**) and LCx (**D**) in the male subjects.

The previously mentioned findings were further confirmed in the male subgroup (Figure 2). A positive correlation was identified between BMI and LAD EAT thickness (r = 0.356, *p* < 0.001), as well as between BMI and LCx EAT thickness (r = 0.300, *p* < 0.001). Notably, in males, age was associated with both LAD EAT thickness (r = 0.308, *p* < 0.001) and LCx EAT thickness (r = 0.335, *p* < 0.001) (Figure 2). These findings support the role of adiposity and aging as key contributors to regional cardiac fat accumulation.

**Figure 3:**
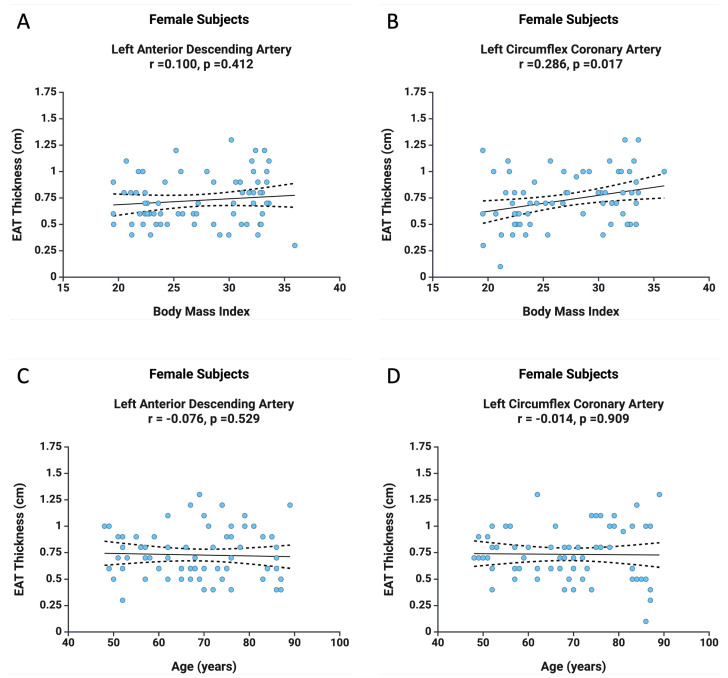
The correlation between BMI and EAT thickness at the level of LAD (**A**) and LCx (**B**), as well as the correlation between age and EAT thickness at the level of LAD (**C**) and LCx (**D**) in the female subjects.

In the female subgroup, a positive correlation was found between BMI and LCx EAT thickness (r = 0.286, *p* = 0.017), but no correlation was noted for LAD EAT thickness (r = 0.100, *p* = 0.412) (Figure 3). Moreover, the relationships observed in all patients, or in the male subgroup specifically regarding age and EAT thickness, were not confirmed in the female subgroup. As illustrated in Figure 3, there is no correlation between age and LAD EAT thickness (r = −0.076, *p* = 0.529) or between age and LCx EAT thickness (r = −0.014, *p* = 0.909). These findings suggest a different pattern of EAT accumulation in females.

**Figure 4:**
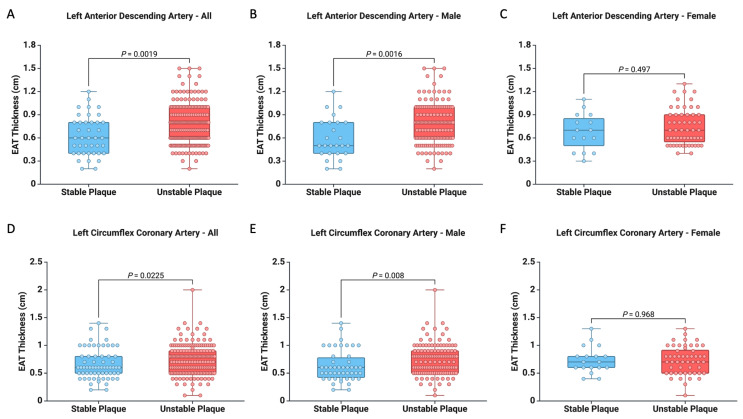
Difference between stable and unstable atherosclerotic plaques in relation to EAT thickness at the LAD artery level across all cases (**A**), within the male subgroup (**B**), and the female subgroup (**C**). Additionally, it presents the disparities concerning plaque vulnerability associated with EAT thickness at the level of the LCx artery for all cases (**D**), the male subgroup (**E**), and the female subgroup (**F**).

Furthermore, we observed a higher EAT thickness at the LAD level (0.77 ± 0.25 vs. 0.62 ± 0.25, *p* = 0.0019) and the LCx level (0.74 ± 0.27 vs. 0.66 ± 0.27, *p* = 0.0225) among subjects with unstable atherosclerotic plaques (Figure 4). These results were also confirmed in the subgroup of male subjects. However, no similar validation was found within the subgroup of female subjects (Figure 4). Consequently, the higher EAT thickness values play a significant role in male subjects in relation to the presence of unstable atherosclerotic plaques.

Further, we analyzed the relationship between age, BMI, EAT thickness, and the presence of unstable atherosclerotic plaque at the LAD and LCX artery levels, utilizing ROC curve analysis. As indicated in Table 2, a good association was observed between EAT thickness at the LAD level and the presence of unstable atherosclerotic plaque in the overall subject population (*p* = 0.003) and within the male subgroup (*p* = 0.001), however, no significant association was found in the female subgroup (*p* = 0.512). Furthermore, we did not identify an association between age, BMI, and the presence of unstable atherosclerotic plaque. In contrast, concerning the presence of unstable atherosclerotic plaque at the LCX level, both age (*p* < 0.001) and EAT thickness at this level (*p* = 0.004) exhibited a significant association (Table 2). These findings were further corroborated in the male subgroup, but no significant association was established within the female subgroup (Table 2).

We employed logistic regression analyses to ascertain whether EAT thickness predicts the presence of unstable atherosclerotic plaque in the LAD and LCx (Table 3 and Table 4). Our findings indicate that elevated values of EAT thickness in the LAD are significantly associated with unstable atherosclerotic plaque (OR: 1.88, 95% CI: 1.26–2.81, *p* = 0.002) for the entire study cohort. Similarly, this association is reflected in the male subgroup (OR: 2.31, 95% CI: 1.35–3.93, *p* = 0.002), while no such association was observed in the female subgroup (Table 3). Furthermore, increased EAT thickness at the LAD is associated with the presence of unstable plaque, independent of demographic variables (OR: 1.80, 95% CI: 1.19–2.73, *p* = 0.005), and remains significant when adjusting for demographic data and BMI (OR: 1.75, 95% CI: 1.15–2.67, *p* = 0.009) (Table 3).

Interestingly, concerning the occurrence of unstable plaque within the LCx, an increased value of EAT thickness serves as an independent predictive factor for the entire subjects enrolled in this study (OR: 1.51, 95%CI: 1.10–2.07, *p* = 0.010) as well as for the male subgroup (OR: 1.83, 95%CI: 1.22–2.74, *p* = 0.003). However, this association loses its statistical significance in the female subgroup and after adjustments for demographic variables and BMI in the entire cohort (Table 4).

## 4. Discussion

The principal outcome of this investigation is the demonstration of the association between the higher value of EAT thickness at the levels of the LAD and LCx and the presence of unstable atherosclerotic plaques within both arterial sites, as observed in a substantial cohort of 245 subjects suffering from SCD. Furthermore, our findings indicate that male individuals are more susceptible to the formation of unstable plaques in the coronary arteries. This indicates that EAT may play a pivotal role in the development and progression of CAD, particularly concerning plaque instability. Despite a statistically significant correlation between BMI and EAT thickness in both arteries, ROC curve analysis revealed that only EAT thickness was significantly associated with unstable plaque. This finding underscores the importance of regional fat distribution, especially in the pericoronary region, as a crucial indicator of coronary pathology rather than exclusively depending on systemic factors such as BMI.

It is well known that higher age and male sex are associated with SCD and play a pivotal role in the development and remodeling of coronary atherosclerotic plaque [21,22,23,24,25]. Recently, Wentzel et al. [21] observed smaller coronary artery diameter and atherosclerotic plaque size in women, but did not observe differences regarding the endothelial shear stress-related plaque progression. However, the authors concluded that plaque progression may be influenced by age within gender. Similar to our study, van Rosendael et al. [22] observed that, in patients under 50 years, only 10% presented calcified plaque, while the incidence of the coronary atherosclerotic burden increases with age.

Concerning the paradoxical role of the BMI to arterial atherosclerotic burden, the presence of unstable plaque, and the occurrence of acute coronary events, a multitude of articles has been published in the literature, yielding mixed results [23,24,25]. Consequently, Senoner et al. [23] conducted a study in which they evaluated cardiovascular risk factors associated with high-risk plaque in a cohort of 1003 patients exhibiting a low coronary artery calcium score (CAC). They noted a positive correlation between obesity and low-attenuation plaque in both female and male participants. Furthermore, Rovella et al. [24] identified a positive correlation between obesity and unstable carotid plaques through multivariate analysis (OR: 5.05, *p* = 0.01). Conversely, studies conducted by Held et al. [25] and Kobayashi et al. [26] corroborate the obesity paradox, revealing that underweight patients are at an increased risk for all-cause mortality and cardiovascular mortality. In the present study, as demonstrated in Table 2, the ROC curve analysis revealed no significant association between BMI and unstable plaque within the left coronary arteries.

The thickness and volume of EAT influence the progression of coronary atherosclerosis severity [12,27,28,29,30,31,32,33,34,35,36], and recent research has highlighted its predictive value in SCD or silent myocardial infarction [15,16]. A meta-analysis by Nerlekar et al. [27], which examined nine studies involving 3772 patients, found that higher levels of EAT correlated with the presence of high-risk plaques (OR: 1.26, *p* < 0.001). Similarly, Djaberi et al. [28] noted that EAT volume predicts coronary atherosclerosis, but not its extent or severity. Additionally, studies have shown that patients with obstructive CAD [12] and asymptomatic CAD patients [32] exhibit increased EAT values determined on coronary computed tomography angiography (CCTA).

Furthermore, Oka et al. [36] observed that an elevated EAT volume is associated with vulnerable components in atherosclerotic plaques. Regarding EAT composition, Kitagawa et al. [37] identified that inflammation and neoangiogenesis correlate with moderate coronary calcification and the formation of non-calcified coronary plaques. Similarly, Khan et al. [38] found that an increased volume of EAT correlates with inflammation and plaque vulnerability. Consistent with the findings of the current study, Lesjak et al. [39] recently published an article indicating that the attenuation of epicardial adipose tissue (EAT) correlates with obstructive coronary artery disease (CAD) in males, but not in females, as determined by multivariable logistic regression analysis. Furthermore, the EPICHEART study identified a relationship between EAT volume and coronary artery calcification (CAC), independent of body size, body fat, or cardiovascular risk factors in men, whereas no such relationship was observed in women [40]. Additionally, Gu et al. [41] reported a higher progression of coronary atherosclerosis in men than in women. Based on the results of the current study and the existing literature [39,40,41], there is a notable prevalence of unstable atherosclerotic plaque in males, correlated with increased EAT thickness, suggesting a potential link between these two factors.

The literature contains scarce information on the comorbidities and risk factors associated with increased EAT thickness and unstable atherosclerotic plaque. Dicker et al. [42] found that hypertensive patients had significantly greater EAT thickness (*p* = 0.011) than normotensive individuals. Furthermore, a meta-analysis conducted by Guan et al. [43], which assessed seven studies, also found elevated EAT levels in patients with hypertension. Several other studies have explored the relationship between type 2 diabetes mellitus (T2DM) and EAT [44,45]. For example, Cetin et al. [44] indicated higher EAT values in T2DM patients compared to controls, revealing a positive correlation between EAT and carotid intima-media thickness. Additionally, Yazıcı et al. [45] confirmed these results within a cohort of type I diabetic patients. These findings suggest that hypertension or diabetes mellitus may have an adverse effect on EAT. However, further research is necessary to establish the causal relationship between these comorbidities and EAT.

Complementing the existing literature, the current study illustrates the predictive role of EAT thickness to the risk of unstable plaque within the left coronary arteries. Moreover, these correlations were predominantly observed in male patients, suggesting the existence of potential sex-based differences in the influence of EAT on coronary artery disease. This may reflect hormonal, metabolic, or anatomical variations between males and females that necessitate further inquiry. Overall, these findings emphasize the potential utility of EAT as a specific marker for plaque instability, particularly within male populations, and underscore the necessity for targeted research concerning gender differences in coronary artery disease risk factors.

### Study Limitations

While we observed significant findings in this study, there are notable limitations to address. Firstly, the participants had a mean age of 62.31 ± 12.69 years, which suggests an elevated risk of atherosclerotic plaque. Moreover, we excluded patients who had died by suicide. Future research should include younger individuals with suicide or drowning as the cause of death to identify early atherosclerotic plaques and examine the involvement of EAT in these cases. Secondly, another limitation is the absence of data concerning the subjects’ comorbidities and risk factors. Another important limitation of the current study is given by the impossibility of analyzing the information regarding the fibrous cap, lipid core, intraplaque neovascularization, inflammation, or other immunohistochemical characteristics of the atherosclerotic plaque. Additionally, we did not measure EAT thickness at the right coronary artery, respectively we couldn’t determine the subjects’ coronary dominance. Lastly, considering the potential for determining the FAI index utilizing CCTA, our findings should be further assessed and confirmed in prospective studies exploring the correlation between EAT thickness and the FAI index and their effects on coronary atherosclerotic plaque remodeling.

## 5. Conclusions

Unstable atherosclerotic plaque significantly contributes to acute coronary events and SCD, even in an apparently healthy population. Nevertheless, current medical practices lack early prognostic tools for the emergence and progression of atherosclerotic plaque, and these plaques are often detected only when patients exhibit symptoms. Our data revealed that higher baseline values of EAT LCx and EAT LAD are associated with unstable plaque at the level of the left coronary arteries. This suggests that EAT could be essential in the onset and advancement of CAD, specifically regarding plaque instability. Although a statistically significant relationship exists between BMI and EAT thickness in both arteries, ROC curve analysis showed that only EAT thickness was notably linked to unstable plaque. This highlights the significance of regional fat distribution, particularly in the pericoronary area, as a vital predictor of coronary disease, rather than solely relying on systemic factors like BMI.

## Figures and Tables

**Table 1:** The age, BMI, and EAT thickness measured at the LAD and LCx levels and the histological type of atherosclerotic plaque in the two coronary arteries are presented for all subjects and by sex.

Variables	All Patients *n* = 245	Male *n* = 175	Female *n* = 70	*p* Value
Age, mean ± SD	62.31 ± 12.69	59.61 ± 12.01	69.07 ± 11.88	**<0.001**
BMI, mean ± SD	26.12 ± 4.16	25.66 ± 3.75	27.28 ± 4.88	0.044
Coronary Artery Characteristics, mean ± SD				
EAT LAD	0.74 ± 0.26	0.75 ± 0.27	0.72 ± 0.23	0.604
EAT LCX	0.71 ± 0.27	0.71 ± 0.28	0.73 ± 0.24	0.377
Histological Type of Left Descending Anterior Artery Plaque, no. (%)				
No plaque	3 (1.22%)	3 (1.71%)	-	0.270
Type I	-	-	-	-
Type II	10 (4.08%)	7 (4.00%)	3 (4.29%)	0.414
Type III	27 (11.02%)	20 (11.43%)	7 (10.00%)	0.138
Type IV	47 (19.18%)	27 (15.42%)	20 (28.57%)	0.018
Type Va	10 (4.08%)	7 (4.00%)	3 (4.29%)	0.919
Type Vb	101 (41.22%)	78 (44.57%)	23 (32.86%)	0.412
Type Vc	20 (8.16%)	17 (9.71%)	3 (4.29%)	0.161
Type VI	28 (11.43%)	25 (14.29%)	3 (4.29%)	0.026
Histological Type of Left Circumflex Coronary Artery Plaque, no. (%)				
No plaque	8 (3.27%)	7 (4.00%)	1 (1.43%)	0.820
Type I	-	-	-	-
Type II	10 (4.08%)	9 (5.14%)	1 (1.43%)	0.184
Type III	46 (18.78%)	31 (17.71%)	15 (25.71%)	0.501
Type IV	79 (32.24%)	53 (30.28%)	26 (37.14%)	0.300
Type Va	6 (2.45%)	6 (3.43%)	-	0.117
Type Vb	50 (20.41%)	33 (18.85%)	17 (24.28%)	0.341
Type Vc	3 (1.22%)	3 (1.71%)	-	0.270
Type VI	43 (17.55%)	34 (19.43%)	9 (12.86%)	0.222
Unstable Atherosclerotic Plaque, no. (%)				
Left Descending Anterior Artery	205 (83.67%)	155 (88.57%)	50 (71.43%)	**0.001**
Left Circumflex Coronary Artery	181 (73.88%)	136 (77.71%)	45 (64.29%)	**0.03**

Statistically significant *p* values are highlighted in bold.

**Table 2:** The characteristics of the ROC Curve concerning age, BMI, and EAT at the LAD and LCx levels, along with their association with unstable atherosclerotic plaque at these locations.

Variables	AUC	Std. Error	95% CI	*p* Value
**Left Anterior Descending Artery—Unstable Plaque**				
Age	0.555	0.058	0.442–0.669	0.338
BMI	0.539	0.049	0.442–0.636	0.426
EAT LAD	0.651	0.050	0.552–0.750	**0.003**
**Left Circumflex Coronary Artery—Unstable Plaque**				
Age	0.685	0.041	0.604–0.767	**<0.001**
BMI	0.541	0.038	0.466–0.616	0.279
EAT LCx	0.621	0.042	0.538–0.704	**0.004**
**Left Anterior Descending Artery—Unstable Plaque—Male**				
Age	0.605	0.073	0.461–0.748	0.153
BMI	0.526	0.056	0.416–0.636	0.644
EAT LAD	0.696	0.061	0.576–0.816	**0.001**
**Left Circumflex Coronary Artery—Unstable Plaque—Male**				
Age	0.752	0.045	0.663–0.841	**<0.001**
BMI	0.547	0.044	0.460–0.634	0.290
EAT LCx	0.666	0.049	0.569–0.763	**0.001**
**Left Anterior Descending Artery—Unstable Plaque—Female**				
Age	0.532	0.094	0.347–0.717	0.734
BMI	0.593	0.092	0.413–0.772	0.311
EAT LAD	0.558	0.088	0.386–0.730	0.512
**Left Circumflex Coronary Artery—Unstable Plaque—Female**				
Age	0.580	0.088	0.408–0.751	0.363
BMI	0.495	0.080	0.338–0.652	0.952
EAT LCx	0.504	0.078	0.351–0.656	0.962

Statistically significant *p* values are highlighted in bold.

**Table 3:** Association between EAT thickness at the LAD level and the risk of unstable atherosclerotic plaque at this level.

Variables	Model	Left Anterior Descending Artery—Unstable Plaque		
		OR *	95% CI	*p* Value
EAT LAD—All	Model 1	1.88	1.26–2.81	**0.002**
	Model 2	1.80	1.19–2.73	**0.005**
	Model 3	1.75	1.15–2.67	**0.009**
EAT LAD—Male	Model 1	2.31	1.35–3.93	**0.002**
	Model 4	2.05	1.20–3.82	**0.009**
EAT LAD—Female	Model 1	1.31	0.72–2.42	0.375
	Model 4	1.29	0.69–2.40	0.421

* OR expressed per 1 SD increase in baseline. Model 1: unadjusted. Model 2: age and sex. Model 3: age, sex, and BMI. Model 4: age and BMI. Statistically significant *p* values are highlighted in bold.

**Table 4:** Association between EAT thickness at the LCx level and the risk of unstable atherosclerotic plaque at this level.

Variables	Model	Left Circumflex Coronary Artery—Unstable Plaque		
		OR *	95% CI	*p* Value
EAT LCx—All	Model 1	1.51	1.10–2.07	**0.010**
	Model 2	1.30	0.91–1.84	0.142
	Model 3	1.24	0.86–1.79	0.249
EAT LCx—Male	Model 1	1.83	1.22–2.74	**0.003**
	Model 4	1.74	1.15–2.64	**0.009**
EAT LCx—Female	Model 1	0.97	0.56–1.66	0.915
	Model 4	0.97	0.55–1.72	0.935

* OR expressed per 1 SD increase in baseline. Model 1: unadjusted. Model 1: age and sex. Model 2: age, sex, and BMI. Model 4: age and BMI. Statistically significant *p* values are highlighted in bold.

## Data Availability

The data that support the findings of this study are available from the corresponding author upon reasonable request.

## Abbreviations

The following abbreviations are used in this manuscript:

CVD	cardiovascular disease
MI	myocardial infarction
EAT	epicardial adipose tissue
MACE	major adverse cardiac events
SCD	sudden cardiac death
ROC	receiver operating characteristic
LAD	left anterior descending artery
LCx	left circumflex coronary artery
